# Severe Postpartum Maternal Morbidity: How Medicaid Coverage for 365 Days Can Make a Difference

**DOI:** 10.7759/cureus.89561

**Published:** 2025-08-07

**Authors:** Lisa B Shields, Angela Glyder, Karisma Chhabria, Clayton Weymouth, Elizabeth Cherot

**Affiliations:** 1 Medical Research and Writing, Lucina Analytics, Boca Raton, USA; 2 Obstetrics and Gynecology, Lucina Analytics, Boca Raton, USA; 3 Data Analytics, Lucina Analytics, Boca Raton, USA; 4 Epidemiology and Public Health, Lucina Analytics, Boca Raton, USA

**Keywords:** medicaid, obstetrics, postpartum, pregnancy-related mortality, public health, severe maternal morbidity

## Abstract

Objective

Severe maternal morbidity (SMM) poses a public health dilemma. To ensure continuity of care for 12 months postpartum, the American Rescue Plan Act of 2021 permitted states to extend Medicaid postpartum coverage to 12 months. This study describes the experiences of a major national insurer in the United States.

Methods

A medical claims and member eligibility dataset was created from a major national insurer over a 10-year period (January 1 2014, to December 31 2023). An individual’s eligibility for Medicaid insurance and ability to enroll in a major national insurer in the United States is based on several factors, including (1) age; (2) income level; (3) number of family members; and (4) whether the individual is pregnant or has a disability. The inclusion criteria in our study consisted of four qualifications: (1) the pregnancy resulted in a delivery; (2) the enrollee retained eligibility with the major national insurer for the full 365 days postpartum; (3) the enrollee did not become pregnant within 365 days postpartum; and (4) the enrollee did not die within 365 days postpartum. The total postpartum episodes refer to any pregnancy that resulted in a delivery. A postpartum episode was labeled as having an SMM when a diagnosis was observed two days prior to the delivery through 365 days postpartum. The timing of SMM was determined according to the first date of service associated with the SMM diagnosis in question. The statistical analysis was performed utilizing Wilcoxon rank sum tests for continuous variables and the chi-squared tests for categorical variables.

Results

Of the 105,654 total postpartum episodes, 3,396 SMM episodes were reported, reflecting 3.2% of the overall number of episodes. The most common SMM conditions were sepsis (999 (29.4%) episodes), acute respiratory distress syndrome (593 (17.5%) episodes), pulmonary edema/acute heart failure (532 (15.7%) episodes), and acute renal failure (531 (15.6%) episodes). A total of 1698 (50%) cases of all SMM presented prior to nine days, and 849 (25%) presented after 131 days. The proportion of those with an older age (29 (IQR = 24-34) years vs. 27 (IQR = 23-32) years), preterm birth (1032 (30.4%) vs. 11,817 (11.6%)), cesarean section (1769 (52.1%) vs. 33,136 (32.4%)), and a depression diagnosis within 12 months postpartum (1081 (31.8%) vs. 20,385 (19.9%)) was higher in individuals with SMM compared to individuals without SMM.

Conclusion

Determining the presence and specific timing of postpartum SMM is critical for intervention by case managers. Expanding Medicaid coverage through the first year postpartum offers a valuable opportunity to monitor healthcare access and manage women with potentially life-threatening pregnancy complications.

## Introduction

The postpartum period serves as an important time for recovering from childbirth, discussing complications of delivery, and transitioning from obstetric to primary care [1]. Severe maternal morbidity (SMM) denotes potentially life-threatening conditions during pregnancy, childbirth, or after pregnancy [2]. According to 2017-2019 data from the Maternal Mortality Review Committee (MMRC) from 36 states in the United States, more than 80% of pregnancy-related deaths are preventable, so it is crucial to identify the etiology and timing of SMM to optimize care delivery [3-5]. The most common SMM observed in a large cross-sectional study of hospital discharges from 2008-2021 were blood transfusion, hysterectomy, acute respiratory distress syndrome (ARDS), acute renal failure, sepsis, eclampsia, shock, and pulmonary edema/acute heart failure [6]. There is limited national data reporting on the timing of these conditions during the perinatal period, despite the fact that SMM is a key indicator and opportunity for risk stratification.

SMM is on the rise in the United States, with significant racial and ethnic disparities [7,8]. Individuals who identified as Black in the National Inpatient Sample were found to have the highest proportion of SMM across the full perinatal period [8]. Individuals who have a lower level of education, those who are African-American or Hispanic, and those with co-existing morbidities, including mental health conditions, have lower rates of postpartum care [9]. The MMRC strongly encourages wider access to healthcare coverage to ensure the continuation of care postpartum.

States are required by federal law to provide pregnancy-related Medicaid (prenatal care, labor and delivery, and postpartum care) to individuals with incomes below 138% of the federal poverty level from conception through the last day of the month in which the 60-day postpartum period ends, after which they may lose coverage [1,10]. To reduce pregnancy-related mortality, SMM, and improve continuity of care for chronic medical conditions (diabetes mellitus, hypertension, cardiac disease, substance use disorder, and depression), a provision in the American Rescue Plan Act permitted states to extend Medicaid postpartum coverage to 12 months through a state plan amendment [11,12]. This Build Back Better Act for postpartum Medicaid expansion was passed by the House of Representatives on November 19, 2021 [1]. It went into effect on April 1, 2022, and was made permanent by the Consolidated Appropriations Act of 2023. As of January 17, 2025, a total of 49 states, including the District of Columbia, had implemented a 12-month extension [12]. One state has a limited coverage extension proposed.

The goal of this study was to present an analysis of SMM in the postpartum period using a large dataset of pregnancies managed by a major national insurer in the United States. The aim was to improve patient outcomes in the context of widespread postpartum Medicaid expansion. The specific SMM episodes and their timing during the postpartum period are presented. We discuss the importance of care coordination in promoting continuity of care and monitoring maternal health for one year postpartum.

## Materials and methods

Study population

Under an Institutional Review Board (IRB)-approved protocol and according to the Declaration of Helsinki, a medical claims and member eligibility dataset was created using data from a major national insurer over a 10-year period (January 1 2014, to December 31 2023). The data in this observational study were collected from 15 states in the United States. An individual’s eligibility for Medicaid insurance and ability to enroll in a major national insurer in the United States is based on several factors, including (1) age; (2) income level; (3) number of family members; and (4) whether the individual is pregnant or has a disability.

Inclusion and exclusion criteria

The inclusion criteria comprised four qualifications: (1) the pregnancy resulted in a delivery; (2) the enrollee retained eligibility in the major national insurer for the full 365 days postpartum; (3) the enrollee did not become pregnant within 365 days postpartum; and (4) the enrollee did not die within 365 days postpartum. The total postpartum episodes refer to any pregnancy that resulted in a delivery.

The exclusion criteria encompassed the following: (1) active postpartum period (has not been 365 days since the delivery); (2) incomplete or missing insurance coverage (the enrollee did not observe or maintain eligibility over the full 365 postpartum days); (3) maternal death within 365 days of delivery due to a postpartum episode (determined by death records from eligibility data or DRG codes indicating death); and (4) the enrollee became pregnant again within 365 days postpartum (activity of pregnancy was observed between 60 and 365 days postpartum, thus indicating a new pregnancy). The total included episodes referred to the total episodes minus the total excluded episodes. Any postpartum episode where a death was observed within 365 days of delivery was not included due to documentation lag that would likely obscure the timeline of SMM.

Data collection

Several metrics were collected in this study: race, maternal age, gestation age at delivery, SMM, cesarean section, use of birth control (long-acting reversible contraception and bilateral salpingectomy), documented depression screen, depression diagnosis, primary care provider visit, emergency room (ER) presentation, and hospital admission. Inpatient hospital admissions were considered when the admit date was between seven and 365 days after delivery; the number of admissions and 30-day readmissions were counted accordingly. ER visits were determined by the Revenue Code 450 or Place of Service Code 23 and were considered when the intake date was between seven and 365 days after delivery; the total number of ER visits and number of visits within 30 days of a previous ER visit were counted accordingly. Postpartum depression comprised both a postpartum or major depression diagnosis within 365 days after delivery. The total number of episodes with depression screens, the number of positive depression screens, and the percentage of follow-up screens within 30 days of a positive screen were also collected. A postpartum episode was labeled as having an SMM when a diagnosis was observed two days prior to the delivery through 365 days postpartum, per the corresponding International Classification of Diseases (ICD) diagnosis code set as outlined by the CDC. The timing of SMM was determined according to the first date of service associated with the SMM diagnosis in question. Five of the 21 indicators of SMM were also coded with the ICD Procedure Coding System (PCS); therefore, they were incompletely reflected in this dataset. These five indicators included conversion of cardiac rhythm, blood transfusion, hysterectomy, temporary tracheostomy, and ventilation.

Statistical analysis

The statistical analysis was performed utilizing Wilcoxon rank sum tests for continuous variables and the chi-squared tests for categorical variables. Box plots were constructed such that each box represented the interquartile range (IQR), with the lower and upper boundaries corresponding to the first and third quartiles, respectively. The line inside each box denoted the median. Whiskers extended to the smallest and largest values within 1.5 times the IQR from the first and third quartiles, respectively. Points that extended beyond the whiskers were considered outliers. Statistical analyses and visualization were conducted using Stata version 17.0 (StataCorp LLC, College Station, TX) [13], R version 4.3.2 (R Core Team, R Foundation for Statistical Computing, Vienna, Austria) [14], and RStudio version 2023.12.1 (Posit, Boston, MA) [15]. A p-value < 0.05 was considered significant.

Ethical approval and informed consent

The WCG IRB determined that this retrospective study was exempt under 45 CFR 46.104(d)(4). The IRB number is 20226602. According to federal regulations, the IRB of record determined that this study was exempt under Category 4 with a complete waiver of consent and authorization.

## Results

Severe maternal morbidity

**Figure 1:**
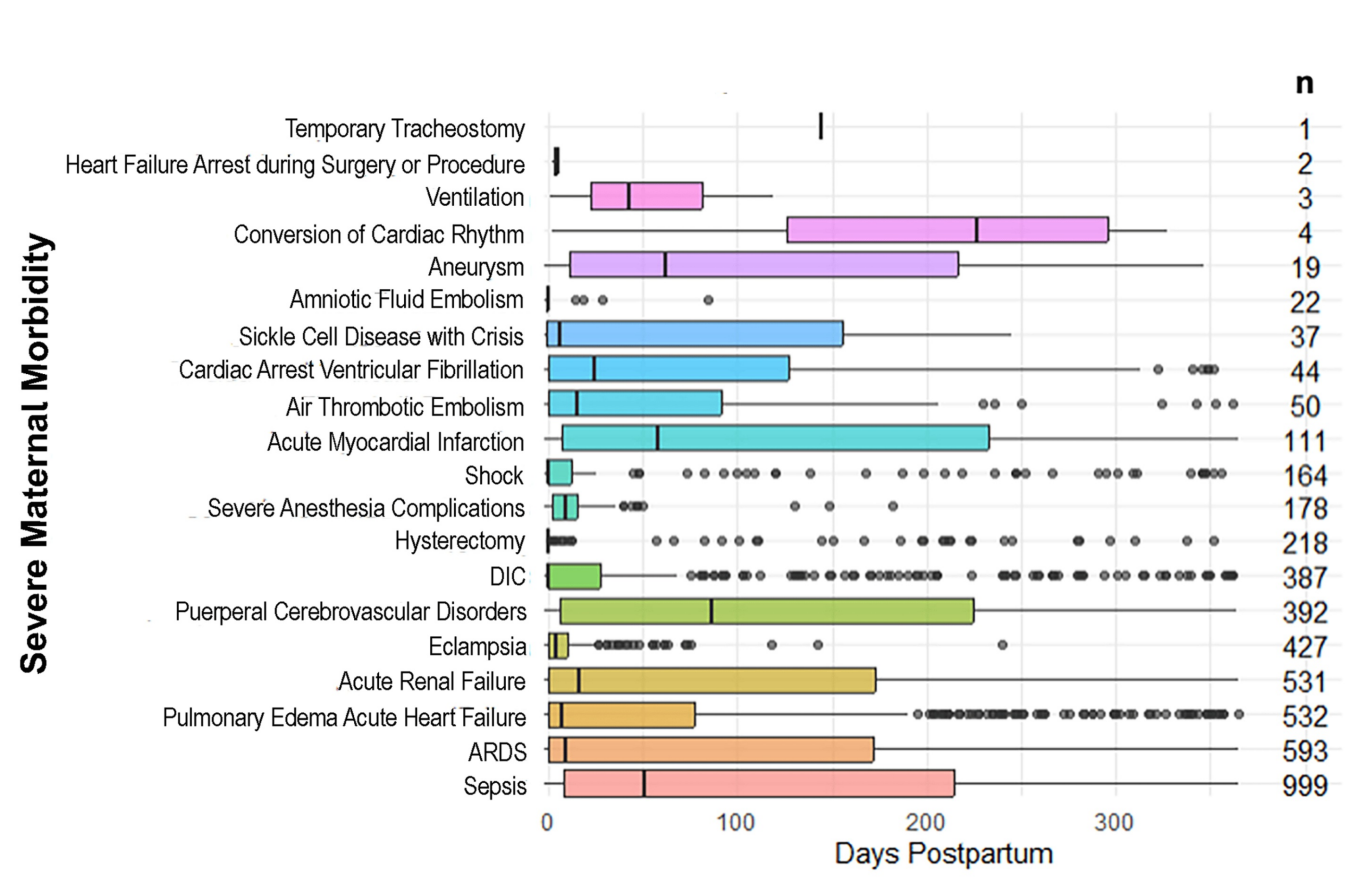
Severe Maternal Morbidity Distribution Over Time During the 365-Day Postpartum Period Each box represents the interquartile range (IQR), with the lower and upper boundaries corresponding to the first and third quartiles, respectively. The line inside each box denotes the median. Whiskers extend to the smallest and largest values within 1.5 times the IQR from the first and third quartiles, respectively. Points that extend beyond the whiskers are considered outliers. The data have been represented as median (IQR). ARDS: acute respiratory distress syndrome; DIC: disseminated intravascular coagulation

Following the implementation of the inclusion and exclusion criteria, a total of 105,654 postpartum episodes were reported over the 10 years of this study (Table 1). A total of 3,396 SMM episodes were reported, reflecting 3.2% of the overall number of episodes. The most common SMM conditions were sepsis (999 (29.4%) episodes), ARDS (593 (17.5%) episodes), pulmonary edema/acute heart failure (532 (15.7%) episodes), and acute renal failure (531 (15.6%) episodes) (Figure 1). Figure 1 depicts the SMM distribution within 365 days postpartum. A total of 1698 (50%) cases of all SMM presented prior to nine days, and 849 (25%) presented after 131 days. Sepsis was the most prolonged, with 250 (25%) of the cases presenting after 214.5 days.

**Table 1:** Characteristics of Pregnancy Episodes With Severe Maternal Morbidity The data have been represented as n, %, and median (IQR). A p-value < 0.05 was considered significant. ER: emergency room; IQR: interquartile range; SMM: severe maternal morbidity; W: Wilcox rank-sum test; χ²: chi-squared test

Demographic characteristics	SMM	Non-SMM	p-value	Test statistic numerical value
	n = 3396	n = 102,258		
	n (%) or median (IQR)	n (%) or median (IQR)		
Race				
American Indian or Alaska Native	20 (0.6)	624 (0.6)	< 0.001	χ² = 96.9
Asian	61 (1.8)	2530 (2.5)		
Black or African-American	1006 (29.6)	23,472 (23.0)		
Hispanic or Latino	495 (14.6)	18,580 (18.2)		
Native Hawaiian or other Pacific Islander	8 (0.2)	196 (0.2)		
Caucasian	1361 (40.1)	43,158 (42.2)		
Other	445 (13.1)	13,698 (13.4)		
Age at year of service (years)	29 (24-34)	27 (23-32)	< 0.001	W = 2.0
Gestation age at delivery (weeks)	40 (36-40)	40 (40-40)	< 0.001	W = 1.45
Gestation age < 37 weeks at delivery	1032 (30.4)	11,817 (11.6)	< 0.001	χ² = 1089.5
Cesarean section	1769 (52.1)	33,136 (32.4)	< 0.001	χ² = 574.9
Birth control within 365 days postpartum	1428 (42.0)	41,292 (40.4)	0.05	χ²= 3.7
Had documented depression screen	510 (15.0)	13,561 (13.3)	0.003	χ² = 8.63
Twelve-month depression diagnosis	1081 (31.8)	20,385 (19.9)	< 0.001	χ² = 286.6
Healthcare utilization				
Presented to primary care	2636 (77.6)	68,273 (66.8)	< 0.001	χ² = 175.0
Average number of primary care visits	2 (1-5)	1 (0-3)	< 0.001	W = 1.1
Presented to the ER	2411 (71.0)	35,395 (34.6)	< 0.001	χ² = 1891.7
Repeat ER visit within 30 days	917 (38.0)	7113 (20.1)	< 0.001	χ² = 433.1
Number of ER visits	2 (1-4)	1 (1-2)	< 0.001	W = 5.7
Admitted inpatient	1267 (37.3)	2925 (2.9)	< 0.001	χ²= 10252.7
Readmitted within 30 days	154 (12.2)	168 (5.7)	< 0.001	χ² = 50.8
Number of inpatient admissions	1 (1-1)	1 (1-1)	< 0.001	W = 2.9
Mean length of stay (days)	3 (2-5)	2 (1.5-4)	< 0.001	W = 2.9

Postpartum episodes with one or more SMM were more likely to be older (29 (IQR = 24-34) years vs. 27 (IQR = 23-32) years), preterm birth (1032 (30.4%) vs. 11,817 (11.6%)), cesarean section (1769 (52.1%) vs. 33,136 (32.4%)), and a depression diagnosis within 12 months postpartum (1081 (31.8%) vs. 20,385 (19.9%)). Postpartum episodes with SMM observed were also more likely to have increased primary care utilization (2636 (77.6%) vs. 68,273 (66.8%)), ER utilization (2411 (71.0%) vs. 35,395 (34.6%)), repeat ER visit within 30 days (917 (38.0%) vs. 7113 (20.1%) of those with ER utilization), inpatient hospitalization (1267 (37.3%) vs. 2925 (2.9%)), and readmission within 30 days (154 (12.2%) vs. 168 (5.7%) of those with inpatient hospitalization). The mean hospital length of stay was longer for those with SMM (3 (IQR = 2-5) vs. 2 (IQR = 1.5-4) days).

Postpartum hospital admissions

A total of 4,192 postpartum episodes had hospital admissions within seven and 365 days after delivery, representing 4.0% of the total number of postpartum episodes (Table 2). Postpartum episodes requiring admission were more likely to be older (28 (IQR = 24-33) years vs. 27 (IQR = 23-32) years), preterm birth (1064 (25.4%) vs. 11,785 (11.6%)), cesarean section (1703 (40.6%) vs. 33,202 (32.7%)), and a depression diagnosis within 12 months postpartum (1733 (41.3%) vs. 19,733 (19.4%)). There was considerable overlap of postpartum episodes requiring admission and those with SMM (n = 1267, 30.2% of postpartum episodes requiring admission, 37.3% of those with SMM). Postpartum episodes requiring admission were also more likely to have increased primary care utilization (3315 (79.1%) vs. 67,594 (66.6%)), ER utilization (3792 (90.5%) vs. 34,014 (33.5%)), and repeat ER visit within 30 days (1660 (43.8%) vs. 6370 (18.7%) of those with ER utilization). A total of 322 (7.7%) of those requiring admission were readmitted within 30 days of discharge. The mean length of stay of inpatient admission was 2.5 days (IQR = 2-4).

**Table 2:** Postpartum Admissions to Inpatient Setting Between Seven- and 365-Day Postpartum The data have been represented as n, %, and median (IQR). A p-value < 0.05 was considered significant. ER: emergency room; IQR: interquartile range; W: Wilcox rank-sum test; χ²: chi-squared test

Demographic characteristics	Postpartum admits	No postpartum admits	p-value	Test statistic numerical value
	n = 4192	n = 101,462		
	n (%) or median (IQR)	n (%) or median (IQR)		
Race				
American Indian or Alaska Native	45 (1.1)	599 (0.6)	< 0.001	χ² = 145.5
Asian	61 (1.5)	2530 (2.5)		
Black or African-American	1236 (29.5)	23,242 (22.9)		
Hispanic or Latino	603 (14.4)	18,472 (18.2)		
Native Hawaiian or other Pacific Islander	6 (0.1)	198 (0.2)		
Caucasian	1715 (40.9)	42,804 (42.2)		
Other	526 (12.5)	13,617 (13.4)		
Age at year of service (years)	28 (24-33)	27 (23-32)	< 0.001	W = 2.3
Gestation age at delivery (weeks)	40 (36-40)	40 (40-40)	< 0.001	W = 1.9
Gestation age < 37 weeks at delivery	1064 (25.4)	11,785 (11.6)	< 0.001	χ² = 715.9
Cesarean section	1703 (40.6)	33,202 (32.7)	< 0.001	χ² = 115.6
Severe maternal morbidity	1267 (30.2)	2129 (2.1)	< 0.001	χ² = 10252.7
Birth control within 365 days postpartum	1786 (42.6)	40,934 (40.3)	0.004	χ² = 8.4
Twelve-month depression diagnosis	1733 (41.3)	19,733 (19.4)	< 0.001	χ² = 1184.9
Healthcare utilization				
Presented to primary care	3315 (79.1)	67,594 (66.6)	< 0.001	χ² = 283.4
Number of primary care visits	2 (1-5)	1 (0-3)	< 0.001	W = 2.8
Presented to ER	3792 (90.5)	34,014 (33.5)	< 0.001	χ² = 5685.3
Repeat ER visit within 30 days	1660 (43.8)	6370 (18.7)	< 0.001	χ² = 1276.6
Number of ER visits	2 (1-4)	1 (1-2)	< 0.001	W = 9.5
Readmitted inpatient within 30 days	322 (7.7)	-	-	-
Number of inpatient admissions	1 (1-1)	-	-	-
Mean length of stay (days)	2.5 (2-4)	-	-	-

Depression diagnosis within 365 days postpartum

A total of 21,466 postpartum episodes had depression diagnoses within 12 months postpartum, reflecting 20.3% of the overall number of postpartum episodes (Table 3). Postpartum episodes with depression diagnoses were more likely to be younger (27 (IQR = 23-31) years vs. 28 (IQR = 23-32) years), preterm birth (3237 (15.1%) vs. 9612 (11.4%)), cesarean section (7671 (35.7%) vs. 27,234 (32.3%)), and SMM (1081 (5.0%) vs. 2315 (2.7%)). Of those with depression diagnoses, 4695 (21.9%) had a documented depression screen, and only 839 (21.8%) of those who screened positive had a timely follow-up screen within 30 days. Postpartum episodes with depression diagnoses were also more likely to have increased primary care utilization (17,956 (83.6%) vs. 52,953 (62.9%)), ER utilization (10,753 (50.1%) vs. 27,053 (32.1%)), repeat ER visit within 30 days (3043 (28.3%) vs. 4987 (18.4%) of those with ER utilization), inpatient admission (1733 (8.1%) vs. 2459 (2.9%)), and readmission within 30 days (183 (10.6%) vs. 139 (5.7%) of those with inpatient hospitalization). The mean length of stay was longer for those with depression diagnoses (3 (IQR = 2-5) vs. 2 (IQR = 1-3.5) days). 

**Table 3:** Depression Diagnosis Within 365-Day Postpartum The data have been represented as N, %, and median (IQR). A p-value < 0.05 was considered significant. ER: emergency room; IQR: interquartile range; W: Wilcox rank-sum test; χ²: chi-squared test

Demographic and clinical characteristics	Twelve-month depression diagnosis	No twelve-month depression diagnosis	p-value	Test statistic numerical value
	n = 21,466	n = 84,188		
	n (%) or median (IQR)	n (%) or median (IQR)		
Race				
American Indian or Alaska Native	201 (0.9)	443 (0.5)	< 0.001	χ² = 3267.5
Asian	258 (1.2)	2333 (2.8)		
Black or African-American	4032 (18.8)	20,446 (24.3)		
Hispanic or Latino	2104 (9.8)	16,971 (20.2)		
Native Hawaiian or other Pacific Islander	25 (0.1)	179 (0.2)		
Caucasian	12,516 (58.3)	32,003 (38.0)		
Other	2330 (10.9)	11,813 (14.0)		
Age at year of service (years)	27 (23-31)	28 (23-32)	< 0.001	W = 1.1
Gestation age at delivery (weeks)	40 (40-40)	40 (40-40)	< 0.001	W = 1.1
Gestation age < 37 weeks at delivery	3237 (15.1)	9612 (11.4)	< 0.001	χ² = 214.4
Cesarean section	7671 (35.7)	27,234 (32.3)	< 0.001	χ² = 88.5
Severe maternal morbidity	1081 (5.0)	2315 (2.7)	< 0.001	χ² = 286.6
Birth control within 365 days postpartum	10,480 (48.8)	32,240 (38.3)	< 0.001	χ² = 786.4
Had documented depression screen	4695 (21.9)	9376 (11.1)	< 0.001	χ² = 1706.5
Timely follow-up screen	839 (21.8)	-	-	
Healthcare utilization				
Presented to primary care, average number of primary care visits	17,956 (83.6); 3 (1-5)	52,953 (62.9); 1 (0-3)	< 0.001 < 0.001	χ² = 3335.9; W = 1.5
Presented to ER	10,753 (50.1)	27,053 (32.1)	< 0.001	χ² = 2400.0
Repeat ER visit within 30 days	3043 (28.3)	4987 (18.4)	< 0.001	χ² = 447.0
Average number of ER visits	1 (1-2)	1 (1-1)	< 0.001	W =
Admitted inpatient	1733 (8.1)	2459 (2.9)	< 0.001	χ² = 1184.9
Readmitted within 30 days	183 (10.6)	139 (5.7)	< 0.001	χ² = 34.2
Average number of inpatient admissions	1 (1-1)	1 (1-1)	< 0.001	W = 3.9
Mean length of stay (days)	3 (2-5)	2 (1-3.5)	< 0.001	W = 4.1

## Discussion

In the current study, the timing of SMM in the 12-month postpartum period is not normally distributed, with a meaningful proportion of cases manifesting after 100 days postpartum. Furthermore, pregnancies with SMM, inpatient admission, and depression diagnoses in the 12 months after delivery were more likely to have increased healthcare utilization and repeat presentation within 30 days. These findings suggest that care coordination can be optimized in this population, especially in light of Medicaid expansion beyond 60 days postpartum [1]. 

Medicaid coverage expansion was driven by the significant public health challenges posed by postpartum pregnancy-related mortality and SMM [16]. Sepsis, ARDS, pulmonary edema/acute heart failure, and acute renal failure were the most frequent SMM conditions in our claims data analysis, consistent with prior literature [6,17]. In Nik Hazlina and colleagues’ systematic review and meta-analysis of risk factors for SMM, risk factors for SMM include cesarean section, advanced maternal age, multiple pregnancies, and co-existing medical conditions [2]. Our study concurs with Nik Hazlina and colleagues’ systematic review and meta-analysis with respect to cesarean birth as a risk factor; however, we were unable to account for existing comorbidities in this cohort. There was overlap across the three classifications (episodes with SMM, inpatient admissions, and depression diagnoses), but there were meaningful proportions of individuals with admissions/ER visits without SMM. 

The postpartum period serves as a valuable time to engage in outpatient services, including longitudinal follow-up, preventative health screenings, and participation in long-term behavioral changes [18]. However, women with pregnancy complications most often miss their postpartum appointments [18]. Care coordination can play an important role during pregnancy and the postpartum period. Clinically experienced care coordinators and managers can dedicate time to thoroughly screen, assess, and comprehend the postpartum needs of each woman, tailoring support based on available resources and assistance. This support includes connecting women to substance use treatment programs, mental health support, contraception, primary care, and lactation support. Additionally, expanded coverage and coordination allow for the important ongoing screening for women with gestational diabetes mellitus (GDM), hypertensive disorders of pregnancy, and/or depression, which comprise a significant proportion of the long-term costs of maternal morbidity [5,9,10,19-21]. Prevention strategies should target not only the new mother but also her family, community, health care provider, health facility, and healthcare system, including medical insurance coverage.

Since the MMRC reported that a high number of pregnancy-related conditions, including SMM, may be preventable, there is an urgent need to intervene both during pregnancy and postpartum to decrease the likelihood of developing a severe medical condition. Women who have recently given birth may fall into a coverage gap or experience a temporary lapse in coverage, which may result in exorbitant out-of-pocket health care costs when they do seek care [10]. Women who suffer from chronic health conditions such as diabetes mellitus or hypertension may face negative health repercussions if their Medicaid coverage is discontinued. By extending postpartum Medicaid coverage to one year after birth, evidence suggests that racial disparities may be curtailed and health outcomes may be improved [10].

Mental health represents a significant cost related to maternal morbidity [21]. Postpartum depression affects approximately 10-15% of women, while postpartum psychosis has a prevalence of one per 1000 births [22]. In Munk-Olsen and colleagues’ study of postpartum mental disorders necessitating hospital admission or outpatient contact for men and women during the one-year postpartum period, these authors reported that primiparous women had an increased risk of hospital admissions with any mental disorders through the first three months after childbirth, with the highest risk at 10-19 days postpartum compared with women who had given birth 11-12 months prior [22]. Additionally, risk was increased for psychiatric outpatient contacts through the first three months postpartum, with the highest risk occurring 10-19 days postpartum. Our study agrees with the reported high likelihood of postpartum depression (10-15% of mothers), although we noted an even higher rate of depression (20.3% of the overall number of episodes) diagnosed within 12 months postpartum. We also noted that postpartum episodes with depression diagnoses had an increased likelihood of SMM as well as increased inpatient care utilization. The high frequency of postpartum depression serves as an important target for care coordinators/managers during the postpartum period, in particular to schedule mental health visits and ensure timely follow-up screenings. Adequately equipping care managers can improve the quality of care in the postpartum setting [23].

It has been reported that women with medically complicated pregnancies have a higher likelihood of ER utilization in the postpartum period [18]. In Harris and colleagues’ study of pregnancies using Medicaid claims data, 20% were complicated by GDM, gestational hypertension, or preeclampsia [18]. Of these complicated pregnancies, 42.1% had GDM, 35.4% had gestational hypertension, and 42.5% had preeclampsia. Women with a pregnancy complication were more likely to have one or more postpartum ER visits during the first six months postpartum compared to women without these complications. The association was highest in women under age 25. Preconception medical comorbidities (type 2 diabetes mellitus, chronic, hypertension, obesity, asthma, mental health problems, and substance abuse) were also strongly associated with postpartum ER use. These authors recommend improved discharge planning and early postpartum care, which may minimize ER use [18]. Additionally, Druyan and colleagues reported that SMM at delivery was associated with an increased risk of inpatient admission in the year following delivery, underscoring the importance of care beyond the traditional six-week postpartum period [17]. A similar finding was noted in the present study, with hospital admissions in 4.0% of all postpartum episodes, which were more common in women with SMM, preterm birth, cesarean section, and depression diagnosis.

Strengths of the current study

The strength of the current work is the large number of claims data over a broad expanse of the United States, reflecting the geographic and racial/ethnic diversity of enrollees. The dataset reflects the experience of a Medicaid managed care organization, which is particularly applicable to the postpartum Medicaid expansion experience nationwide. The years of our study were unique in that they spanned a time period when Medicaid initially had temporary postpartum coverage and then implemented postpartum expansion.

Limitations of the current study

Limitations include the retrospective nature and the use of claims data with the associated documentation lag, retroactive diagnoses, and incomplete representation of conditions relying on the ICD-PCS code set, which was not available in our dataset. The lack of retail prescription claims and documentation of existing comorbidities also prevented adequate risk adjustment when comparing cohorts, especially in the context of a dynamic policy landscape. Due to the claims abstraction methodology, we were unable to include individuals with interpregnancy intervals less than 365 days or those who had maternal mortality, a group suspected to have high rates of SMM. Another limitation of this study is that the enrollees qualified and retained coverage for the full 365 days postpartum. Under the American Rescue Plan Act, women who previously did not qualify by income requirements will now retain coverage, potentially introducing differences in medical experiences compared to those historically covered. Another limitation is that five indicators of SMM were also coded with the ICD PCS, and therefore, they were incompletely reflected in this dataset. These five indicators included conversion of cardiac rhythm, blood transfusion, hysterectomy, temporary tracheostomy, and ventilation. Future studies should explore any differences among the newly covered populations to complement our findings.

## Conclusions

Ascertaining the presence and specific timing of postpartum SMM presents a valuable opportunity for care coordinators/managers to provide care coordination to postpartum women. Extending Medicaid coverage during the initial postpartum year presents the opportunity for women to maintain ongoing monitoring and ensure consistent access to healthcare services. Through sustained coverage and healthcare access, screening and management can be enhanced for women at risk of life-threatening pregnancy complications, thereby mitigating maternal morbidity and mortality rates. Further investigation into postpartum health is warranted, with a focus on newly insured populations as well as specific interventions that improve outcomes in high-risk groups. Additional studies are needed to identify drivers of inpatient healthcare utilization as well as strategies to ensure that individuals with SMM are supported in the outpatient setting through one full year postpartum.
